# Captive Bottlenose Dolphins (*Tursiops truncatus*) Spontaneously Using Water Flow to Manipulate Objects

**DOI:** 10.1371/journal.pone.0107796

**Published:** 2014-09-24

**Authors:** Chisato Yamamoto, Keisuke Furuta, Michihiro Taki, Tadamichi Morisaka

**Affiliations:** 1 Graduate School of Fisheries Science and Environmental Studies, Nagasaki University, Nagasaki, Japan; 2 Kobe City Suma Aqualife Park, Kobe, Japan; 3 Miyajima Public Aquarium, Hatsukaichi, Hiroshima, Japan; 4 Wildlife Research Center of Kyoto University, Sakyo-ku, Kyoto, Japan; 5 Institute of Innovative Science and Technology, Tokai University, Shimizu-ku, Shizuoka, Japan; Universität Bielefeld, Germany

## Abstract

Several terrestrial animals and delphinids manipulate objects in a tactile manner, using parts of their bodies, such as their mouths or hands. In this paper, we report that bottlenose dolphins (*Tursiops truncatus*) manipulate objects not by direct bodily contact, but by spontaneous water flow. Three of four dolphins at Suma Aqualife Park performed object manipulation with food. The typical sequence of object manipulation consisted of a three step procedure. First, the dolphins released the object from the sides of their mouths while assuming a head-down posture near the floor. They then manipulated the object around their mouths and caught it. Finally, they ceased to engage in their head-down posture and started to swim. When the dolphins moved the object, they used the water current in the pool or moved their head. These results showed that dolphins manipulate objects using movements that do not directly involve contact between a body part and the object. In the event the dolphins dropped the object on the floor, they lifted it by making water flow in one of three methods: opening and closing their mouths repeatedly, moving their heads lengthwise, or making circular head motions. This result suggests that bottlenose dolphins spontaneously change their environment to manipulate objects. The reason why aquatic animals like dolphins do object manipulation by changing their environment but terrestrial animals do not may be that the viscosity of the aquatic environment is much higher than it is in terrestrial environments. This is the first report thus far of any non-human mammal engaging in object manipulation using several methods to change their environment.

## Introduction

Object manipulation in animals is defined as any physical touching with the object except for incidental touching [1]. Various types of object manipulation in animals have been reported such as rubbing, dropping, pulling, throwing, and stacking [1], [2]. The ability to manipulate objects is believed to have provided a base for tool use in animals [3].

Various object manipulations using the mouth or hands are present in terrestrial animals, especially in primates. For example, chimpanzees (*Pan troglodytes*) walk backwards while pulling many dry leaves along the ground with both hands [4]. Bonobos (*Pan paniscus*) rub a substrate or their body with an object in their hands, or hold it in one hand and put it in their mouths [5]. Japanese macaques (*Macaca fuscata*) repeatedly pick up stones and drop them, or carry them by hand from one place to another [6]. Domestic cats (*Felis silvestris catus*) hit objects with their front paws, or hold and lightly bite them in their mouths [7]. Neotropic cormorants (*Phalacrocorax brasilianus*) hold sticks or fish in their bills and plunge them into the water, or toss fish into the air using quick, upward head movements [8].

Delphinids also show object manipulation, both in the wild and in captivity. They use body parts, such as their mouths or fins, and several detailed studies have addressed these behaviors. For example, bottlenose dolphins (*Tursiops truncatus*) use their rostrums to toss food [9], their heads to balance kelp, and their tails to flip food to their dorsal fins [10]. Rough-toothed dolphins (*Steno bredanensis*) hold a piece of plastic in their mouth, drop it, and receive it with their pectoral fins [11]. Dusky dolphins (*Lagenorhynchus obscurus*) carry kelp using their rostrums, melons, flippers, and dorsal fins [12].

In most previous studies of terrestrial animals and delphinids, animals manipulate the object using their body parts. In contrast, we here report on captive bottlenose dolphins spontaneously manipulating objects using water flow made by their movements. Object manipulation including tool use has received attention from a viewpoint of cognition, especially understanding of physical phenomena in animals [2], [13]. Use of water flow without distinct adaptive value (like feeding) reported here may relate an understanding of the physical phenomena.

## Materials and Method

### Ethics statement

This study complied with the “WAZA Ethical Guidelines for the Conduct of Research on Animals by Zoo and Aquariums.” Research permission for this study was granted by Kobe City Suma Aqualife Park, Japan. This study was observational and did not involve the handling of animals. Our observation did not affect the dolphins' welfare.

### Subjects and facility

Four captive bottlenose dolphins (*Tursiops truncatus*) in Kobe City Suma Aqualife Park in Hyogo, Japan, were the subjects of our study. They comprised one juvenile male, “Smile”, and three adult females, “Coo”, “F1”, and “Mammy” (Table 1). Smile was born in Suma Aqualife Park, but none of the three females was his mother. All dolphins lived in the performance pool (20 m major axis, 13 m minor axis, 3.5 m deep).

**Table 1:** Sex, age, and year that dolphins were born or year of arrival.

	Sex	Age	Year of Birth or Arrival
Coo	Female	13 (estimated)	Since 2004
F1	Female	18 (estimated)	Since 1995
Mammy	Female	12 (estimated)	Since 2004
Smile	Male	5	Born in Suma Aqualife Park in 2000

### Data collection

Behavioral data were collected for nine days in total in November 2005. Visual observations were made between 9:00 and 17:00 from the underwater window (1 m×1.5 m, 0.95 m from the water surface). All dolphins had six 30-min feeding times per day, with the exception of seven times on Sunday. We did not observe them during feeding time.

From preliminary observations, we found the typical sequence of object manipulations with food. First, dolphins approached the floor and assumed a head-down posture, and then released the food from their mouths. They then moved the food around their mouths and caught it again, or they caught it without moving it. Dolphins sometimes dropped their food on the floor, failing to catch it, and then picked it up again. Finally, dolphins ceased assuming the head-down posture, and began to swim.

Because dolphins repeatedly released their food and moved it around their mouths, we defined the period of object manipulation as between when dolphins assumed a head-down posture and when they began to swim once more. To investigate the pattern of object manipulation, we recorded the object types (fish or squid) used in object manipulation, places where dolphins first released the object from their mouths, and places where they moved the object after releasing it. To investigate how dolphins manipulated the object, we recorded the method they moved it and how they picked up the object once it had been dropped.

### Statistical analysis

To investigate whether the pattern and manner of object manipulation differed between individuals, we used Fisher's exact test with Bonferroni correction (0.05/3 = 0.02) for two analyses as follows: (1) the ratio of times the object was placed at each location was compared between individuals, and (2) a comparison of the ratio of times dolphins used each different method to pick up the object. To test whether object types affected the pattern and manner of object manipulation, we conducted the following analyses using Fisher's exact test for each individual: (a) the ratio of bouts using each object type (fish and squid), (b) the ratio with which dolphins placed objects at each location, and (c) the ratios of each method used to pick up each object type. R 2.14.0 was used for statistical analysis.

## Results

### The number and duration of object manipulation

There were 407 bouts of object manipulation used for behavior analyses (245 for Coo, 132 for Mammy, 30 for Smile). F1 did not perform any object manipulation with food throughout the study period. The average duration of a bout of manipulation was 41.0 s (SD = 14.6) for Coo, 44.5 s (SD = 20.2) for Mammy and 28.1 s (SD  = 13.6) for Smile.

### Food type using object manipulation

Coo and Mammy manipulated both fish and squid, but Smile manipulated only fish. The number of times that Coo manipulated the squid was significantly greater than the number of times she manipulated the fish (Fish, 22 times; Squid, 223 times; Fisher's exact test, d.f. = 1, *P*<0.001), whereas the number of times that Mammy manipulated the fish was significantly greater than the number of times she manipulated the squid (Fish, 90 times; Squid, 42 times; Fisher's exact test, d.f. = 1, *P*<0.001).

### Object release location and placement area

**Figure 1:**
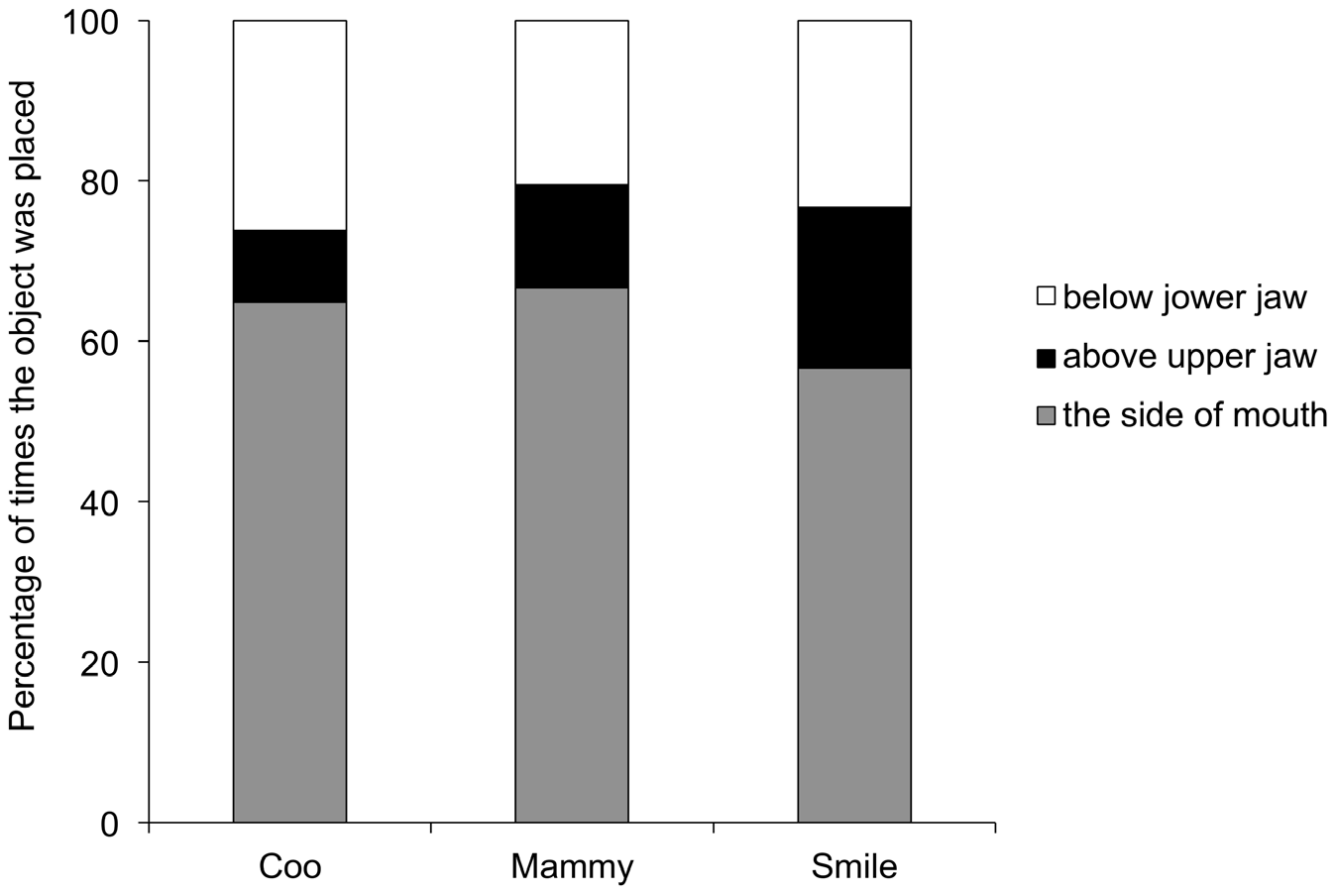
Object placement at each location. The ratio of object placement at each location for the total observed bouts for each individual. The legend indicates the location at which the object was placed. Placement to the side of the mouth indicates no object manipulation and placement below the lower jaw and above the upper jaw indicate object manipulation.

Dolphins were able to release the object from a position that could be classified both as the front and as the side of their rostrums. All dolphins performing object manipulation always released the object from the sides of their mouths. The three places where dolphins placed the food after releasing it from the sides of their mouths were observed as follows: (a) dolphins placed the object on the sides of their mouths (they did not move the object), (b) dolphins moved and placed the object above their upper jaws, and (c) dolphins moved and placed the object below their lower jaws. The ratio at which the object was placed at each location did not differ between individuals (Fisher's exact test with Bonferroni correction, d.f. = 2, Coo vs. Smile: *P* = 0.20, Coo vs. Mammy: *P* = 0.29, Smile vs. Mammy: *P* = 0.48, Fig. 1). For Coo and Mammy, who used both fish and squid for object manipulation, object types did not affect the ratio with which they placed the object at each location (Fisher's exact test, d.f. = 2, Coo: *P* = 0.25, Mammy: *P* = 0.15).

### Object manipulation after release

We observed two methods that the dolphins moved the object from the side of their mouths to above the upper jaw or below the lower jaw, including: (a) dolphins did not show physical movement, but the object was slowly moved by the water current in the pool, and (b) dolphins moved their head, then the object quickly moved around the dolphin's mouth. We observed that all dolphins used both methods, but accurate frequency measurements were not recorded.

### Object retrieval after is drop

**Figure 2:**
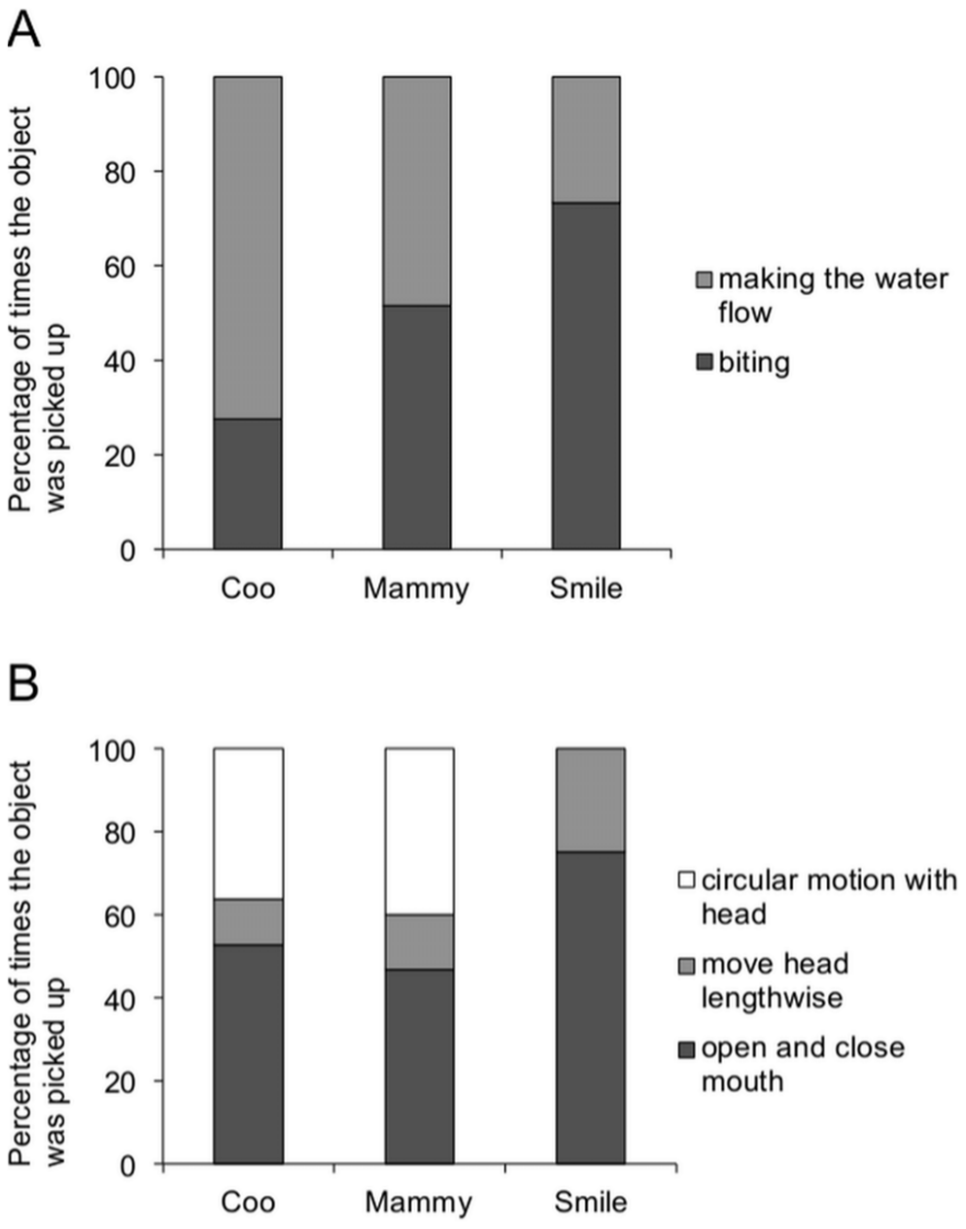
Object pick-up method. (A) The ratio of biting or making the water flow when picking up the object in relation to the total number of times it was dropped. (B) The ratio of each method of creating water flow out of the total number of times the water flow was created for each individual. The legend indicate the method used to make the water flow.

Dolphins showed two methods of picking up the object that they had dropped on the floor: (a) dolphins directly bit the object on the floor, and (b) dolphins moved their bodies near to the object, then lifted the object from the floor. When the dolphins did not move their bodies, the object remained still on the floor (Video S1), which indicates that dolphins' movements lifted the object using water flow. All dolphins employed both methods. The ratio of use of each pick up method was not significantly different between Coo and Mammy or between Mammy and Smile (Fisher's exact test with Bonferroni correction, d.f. = 1, Coo vs. Mammy: *P* = 0.02, Mammy vs. Smile: *P* = 0.21, Fig. 2A). However, the ratio of water flow use for Coo was higher than it was for Smile (Fisher's exact test with Bonferroni correction, d.f. = 1, *P* = 0.001, Fig. 2A). For Coo and Mammy, who used two object types, the ratio of each method's use did not differ between object types (Fisher's exact test, d.f. = 1, Coo: *P* = 0.10, Mammy: *P* = 0.48).

Three different method of making water flow were observed: (a) dolphins opened and closed their mouths repeatedly towards the object, (b) dolphins moved their heads lengthwise towards the object, and (c) dolphins made circular motions with their heads towards the object. Coo and Mammy exhibited all three methods, but Smile did not perform circular head movements. There were no differences in the ratio of each method's use between Coo and Mammy or between Mammy and Smile (Fisher's exact test with Bonferroni correction, d.f. = 2, Coo vs. Mammy: *P* = 0.14, Mammy vs. Smile: *P* = 0.33, Fig. 2B), although a significant difference was noted between Coo and Smile (Fisher's exact test with Bonferroni correction, d.f. = 2, *P* = 0.002, Fig. 2B). For individuals who used two object types, object type did not affect the method use ratio (Fisher's exact test, d.f. = 1, Coo: *P* = 0.33, Mammy: *P* = 0.06).

## Discussion

We describe our observations of bottlenose dolphins spontaneously changing their environment and employing water flow to manipulate objects. Manipulating objects using the mouth is common in terrestrial animals and delphinids (e.g., primates [1], New Caledonian crows; *Corvus moneduloides* [14], and delphinids [15]). However, object manipulation using physical power (such as gravity, buoyancy, or flow) has been reported in few studies. Common ravens (*Corvus corax*) have been found to release objects in the air, allowing gravity to lead them to fall, and catching them before they touch the ground [16]. Chimpanzees (*Pongo abelii*) have been found to use buoyancy in object manipulation [1]. They floating objects on water and poking them with their fingers. Bottlenose dolphins have been showed to use flow during manipulating an object. They released a feather into a water jet and retrieved it after water flow had pushed the feather away [17].

Our observations, on the other hand, demonstrate that dolphins actively and spontaneously change their environment, making water flow to engage in object manipulation. Few studies have shown spontaneous aquatic animal-instigated environment change for object manipulation. Some species, such as south american fresh water stingrays (*Potamotrygon castexi*) [18], arabesque greenlings (*Pleurogrammus azonus*) [19], and killer whales (*Orcinus orca*) [20] have demonstrated spontaneous animal-instigated environmental change. In all of these studies, however, the animals used just one stereotyped method of creating water flow to manipulate their environment. In contrast, our dolphins made water flow using several flexible methods. The reason that only aquatic animals show spontaneous change of the environment may be that the viscosity of the aquatic environment is much higher than that of the terrestrial environment. The power transmitted by the animal's movement is lager when the viscosity is higher than when it is lower. Therefore, aquatic animals may change their environments more easily than terrestrial animals.

Cetaceans use water to manipulate objects as well, especially during feeding. For example, humpback whales (*Megaptera novaeangliae*) use bubbles during feeding, which is called bubble-net feeding [21]. This feeding method differs across sea areas and individuals [21], [22]. In this case, they control living prey's behavior by encircling the prey with a bubble net, which is automatically created by breathing out. In contrast, our study on dolphins showed two manipulation behaviors to move inanimate objects. First, a water flow was created by an active dolphin's movement and then this water flow moved the object. Here, the reported behavior that inanimate objects are moved by water flow differed from bubble-net feeding that behavior of living prey is controlled.

Another form of using water to manipulate objects during foraging is suction feeding. Odontocetes draw their prey by increasing the volume of their oral cavity [23]. Suction force is higher in odontocetes that can shorten and widen their rostrum and jaws [24]; thus, suction feeding ability is dependent on morphological features. Suction feeding in odontocetes was suggested to evolve as the way that pray are grasped by long jaw and are transported the oral cavity by suction, then the way that prey are caught by suction [25]. In terms of using water current, suction feeding behavior is similar to the water flow object manipulation made by the dolphins' movements. However, the difference is that suction feeding evolve as way to eat prey [25]. In contrast, object manipulation of our study did not feeding behavior and our results suggest that dolphins have learned to pick up the object using water flow (see below). This is the first report of any mammal, except humans, manipulating objects using several methods of changing their environment spontaneously.

The three dolphins performed similar types of object manipulations with their food. The sequence of object manipulation was the same for all dolphins. Dolphins showed a head-down posture during object manipulation. They always released the object from the side of the mouth and manipulated it around the mouth. The methods of manipulation were similar for all individuals: indeed, all dolphins used two techniques when moving the object, and two of the three techniques to make the water flow when picking up the object. The ratio of placements at each location out of the total number did not differ between individuals. There were no significant differences in the ratio of using water flow to pick up the object and the ratio of methods used to make water flow between individuals, except for one pair. The object type did not affect the locations where the dolphins placed the object after releasing it, the method of picking up the object up, or the method of making the water flow to pick up the object.

Matsusaka et al. [26] suggested that social learning played an important role in the spread of behavior within the group, and bottlenose dolphins were previously suggested to have some capacity for social learning [27]. In this study group, the result that object manipulation methods are similar in three dolphins may be possible that some dolphins initiated object manipulation with food and this behavior spread to other dolphins. One dolphin that did not manipulate the object, F1, may be an example of this, but further research regarding learning is needed to investigate the behavior of individual dolphins that do not manipulate.

Our study had several limitations. First, since we observed only one group, we could not demonstrate whether bottlenose dolphins generally produce water flow to manipulate objects, and the types of situations that elicited such behavior. To do so, further research should be conducted on additional dolphin groups, including a wild population, and examine the circumstances surrounding water flow use. Second, we did not observe the process of acquiring the object manipulation technique using water flow. Therefore, we cannot ascertain whether dolphins had understood that their movements made the water flow, or had learned to associate their behavior (i.e., moving the head) with the result (i.e., moving the object). Further study is therefore needed to assess whether bottlenose dolphins understand the physical properties of object manipulation.

## Supporting Information

Dataset S1 **Dataset included in analyses.**
(XLSX)

Video S1 **Object manipulation by bottlenose dolphins in Suma Aqualife Park.** We recorded video from 12–13 April, 2014. Bottlenose dolphins manipulated the object around their mouth. The object was afloat when dolphins moved near, and this was not due to the general water current in the pool.
(MOV)
